# Artificial sweeteners and their implications for patients with diabetes: a systematic review and meta-analysis

**DOI:** 10.25122/jml-2025-0135

**Published:** 2026-01

**Authors:** Adrian Lück, Fabian Standl, Heribert Stich

**Affiliations:** 1 Pettenkofer School of Public Health, Munich, Germany; 2 Department of Public Health Medicine, Augsburg, Germany; 3 Technical University of Munich, Germany, TUM School of Medicine and Health, Graduate Center of Medicine and Health, Munich, Germany; 4 Institute for Medical Processing, Biometry, and Epidemiology-IBE, University of Munich-LMU, Munich, Germany; 5 Department of Public Health Medicine, Landshut, Germany

**Keywords:** artificial sweeteners, non-nutritive sweeteners, diabetes, PRISMA, systematic review, meta-analysis, ADI, Acceptable Daily Intake, AS, Artificial sweeteners, CI, confidence interval, EFSA, European Food Safety Authority, MeSH, Medical Subject Headings, PRISMA, Preferred Reporting Items for Systematic Reviews and Meta-Analyses, RCT, randomized controlled trials, SD, standard deviation, SE, standard error, FDA, US Food and Drug Administration, WHO, World Health Organization

## Abstract

Since their discovery in the 18^th^ century, artificial sweeteners have been an important part of modern-day nutrition. The same applies to sugar consumption, which has increased massively in the last few decades. That is why the World Health Organization (WHO) recommends limiting free sugar intake to less than 10% of total daily energy intake, with additional benefits likely at levels below 5% per day. This high consumption led to the fact that diabetes is now one of the most frequent comorbidities in the world, with around 529 million people suffering from this disease. Patients need to be careful with their diet, which raises the question of whether artificial sweeteners should be mandatory in a diabetic nutrition plan. To answer this question, a systematic review following PRISMA guidelines and a meta-analysis were conducted. Overall, 22 studies were included in this review. The results of the studies were ambivalent, and no general statement could thus be made. We found the same results in the meta-analysis, where Hedges' g was used as an effect size. Artificial sweeteners were associated with higher insulin (g = 0.50, 95% CI, 0.19-0.82) and higher HbA1c (g = 0.30, 95% CI, 0.06-0.54) in common-effects models; however, these associations were not relevant in random-effects models, and heterogeneity was substantial. No consistent effects were observed for other outcomes. Based on current evidence, the routine use of artificial sweeteners in diabetic diets cannot be recommended.

## INTRODUCTION

Rising sugar intake is a problem for health services worldwide. From 1990 to 2018, the consumption of sugar-sweetened beverages increased by 23% in 185 countries [1]. The consequence of this is the rising prevalence of obesity, cardiovascular diseases, and type 2 diabetes [2]. The World Health Organization (WHO) recommends limiting free sugar intake to less than 10% of total daily energy intake, with additional health benefits expected at levels below 5% [3]. For a regular adult, this is roughly 50g of free sugar per day. A higher consumption, especially for people with diabetes, is potentially harmful. One way to reduce the consequences of diabetes is through a diet. Artificial sweeteners (AS) could help within a specific diet plan. Using sweeteners has two major benefits: they have fewer calories than regular sugar, and they are sweeter.

In the United States, the U.S. Food & Drug Administration (FDA) approved six AS: aspartame, acesulfame potassium, sucralose, neotame, advantame, and saccharin [4]. Those substances have an acceptable daily intake (ADI), which represents the maximum dose of sweeteners a human can consume before getting adverse effects. These AS are also legal in Europe, but are currently under reinvestigation from the European Food and Safety Authority (EFSA) due to new health concerns [5].

Despite the approval, the WHO developed a guideline in 2013 to generate stronger scientific evidence [6]. They reviewed 50 randomized controlled trials (RCTs), 97 prospective cohort studies, and 41 case-control studies. They found that a long-term intake of AS led to a higher body weight, increased the risk of type 2 diabetes and cardiovascular diseases, and a higher risk for preterm birth in pregnant women [6]. Those insights led to the conclusion that the WHO does not recommend the use of AS for weight control or for decreasing the risk of noncommunicable diseases [6]. This review and meta-analysis focused on whether AS could be beneficial for patients with diabetes and whether they should be included in specific diet plans despite evidence of adverse effects.

## MATERIAL AND Methods

### Searches

A systematic review and meta-analysis were performed between October 2024 and January 2025. The literature search was conducted according to the PRISMA guidelines developed by Page *et al*. [7]. Four databases were used for the systematic review: PubMed, Cochrane Library, Embase (via Ovid), and Web of Science. A search string was generated using suitable keywords from the keyword index in the different databases. The selected keywords and their synonyms were categorized into three groups: AS, diabetes, and outcomes. A detailed list of the keywords used is provided in Supplementary Table S1. Within each group, different synonyms were combined using Boolean operators. In PubMed, the Medical Subject Headings (MeSH) vocabulary was also used to obtain more detailed search results. Grey literature (e.g., Google Scholar) was not searched due to the substantial number of records retrieved from the selected databases.

Supplementary File

### Study inclusion and exclusion criteria

The inclusion and exclusion criteria were determined using the PICOS (Population, Intervention, Comparator, Outcomes, and Study design) framework, an extended version of the PICO framework that includes the study design component [8]. A summary of all criteria is provided in Supplementary Table S2. Eligible studies were required to focus on human adults (≥18 years) with any form of diabetes. Studies with animals, children, or healthy participants (unless in the control group) were excluded. In addition, the intervention had to involve AS and no natural sweeteners, such as sugar or honey. The control groups in the included studies were required to compare AS with other dietary interventions (e.g., sugar or low-calorie diets) or with healthy adults. All included studies were required to report diabetes-related outcomes (e.g., blood glucose levels, glycated hemoglobin [HbA1c], or weight management outcomes), with no focus on participants' taste or preferences. RCTs, cohort studies, case-control studies, and cross-sectional studies were included in this review, while all other study types were excluded. Eligible studies had to be published in English between 2004 and October 2024. No distinction was made between diabetes treatment regimens (e.g., insulin vs. oral antidiabetic therapy), which may contribute to variability in metabolic outcomes across studies. The type of diabetes (type 1, type 2, or latent autoimmune diabetes in adults [LADA]) was recorded when available but was not used as an inclusion criterion, as many studies reported mixed or unspecified populations. Body mass index (BMI) was not used as an eligibility or adjustment criterion due to inconsistent reporting across studies.

### Study quality assessment, data extraction strategy, data synthesis, and presentation

RCTs were assessed using the Cochrane Risk of Bias Tool [9]. Data extraction was performed by a single reviewer, which may introduce selection or classification bias. Cohort and case-control studies were evaluated using the Newcastle–Ottawa Scale [10]. The quality of cross-sectional studies was assessed using the Joanna Briggs Institute (JBI) Critical Appraisal Checklist [11]. The effect measure used in this meta-analysis was Hedges’ g. This measure is suitable for this type of analysis because it statistically adjusts the variance that may occur when sample sizes are small [12]. Another advantage of using Hedges' g as an effect size is that it allows data synthesis across different levels of measurement. This is useful because outcomes are measured differently [13]. Sensitivity analyses (e.g., leave-one-out) were not conducted due to the limited number of studies available per outcome.

## Results

### Review statistics

**Figure 1:**
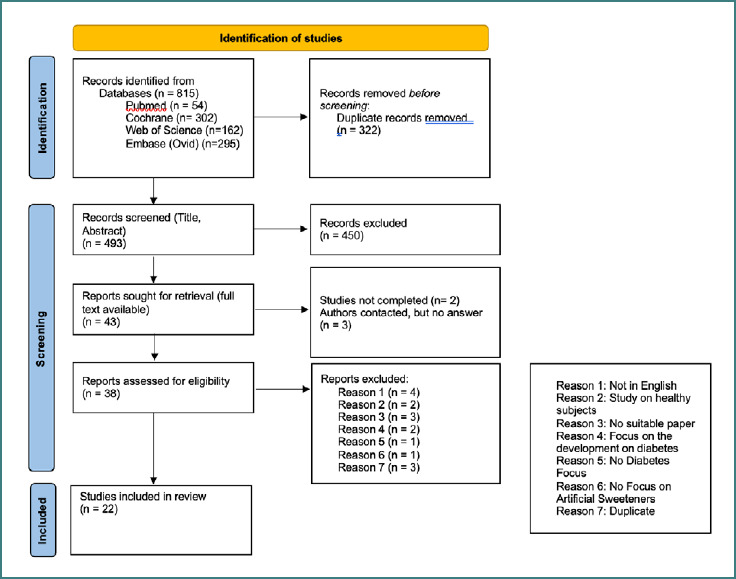
Preferred reporting items for systematic review and meta-analysis (PRISMA) [7]

The detailed search process is shown in Figure 1. Overall, in all four databases, 815 articles were found. After removing the duplicates (*n* = 322), 493 articles remained for the title and abstract screening. 450 papers were excluded, and 43 were suitable for the full-text screening. Two studies were not completed at this point, and the full texts of three studies were unavailable. In total, 38 articles were assessed for eligibility. 16 studies were excluded for the following reasons: not in English, study on healthy subjects, no suitable paper, focus on diabetes development, no focus on diabetes, no focus on artificial sweeteners, duplicate. 22 studies remained and were included in this systematic review and the meta-analysis. The studies included in this review comprise 12 RCTs, 7 cross-sectional studies, 2 case-control studies, and 1 cohort study, resulting in an overall sample of 6,969 participants. The results of the quality assessment process are shown in Supplementary Table S3. All included RCTs demonstrated a low to moderate risk of bias and were therefore considered eligible for inclusion in the analysis. The same applied to the case-control and cohort studies. In contrast, issues were identified in the cross-sectional studies: none were rated as having a low risk of bias; instead, their risk ranged from moderate to high. These studies had a relatively high number of methodological limitations. The results of those articles will be reported, but the high risk of bias will be taken into account in the interpretation.

In summary, the RCTs showed heterogeneous outcomes for relevant diabetic parameters, and no clear conclusions can be drawn. A similar inconsistency was observed in lipid profile outcomes: some studies reported that consumption of AS was associated with increased levels of low-density lipoprotein (LDL), high-density lipoprotein (HDL), and triglycerides, whereas Ajami *et al*. [14] reported a reduction. The results for weight loss and diet quality were also ambiguous. Cross-sectional studies indicated that people, especially in rural areas, lacked precise knowledge of AS and its potential consequences for their bodies. The results could also link the consumption of AS with hypertension and obesity. The results of the case-control/cohort studies concluded that the consumption of sweetened beverages was linked to a risk of LADA or type 2 diabetes [15] and showed a 1.6 times higher risk of gestational diabetes mellitus (GDM) for women who consumed any AS [16].

### Study quality assessment

Level 1a evidence, the focus of the meta-analysis was laid on the 12 RCTs. Those were identified during the systematic review. Overall, eight outcomes were included in the calculation: insulin, glucose, C-peptide, glucagon-like peptide-1 (GLP-1), LDL, HDL, triglycerides, and HbA1c. Systolic and diastolic blood pressure were not included in the analysis, as only one study reported these outcomes. All the results were summarized in a single forest plot (Figure S1, Supplementary file). The numerical results of the meta-analysis are presented in Table 1, while the results of the Egger test for publication bias are summarized in Table 2. For every outcome, a detailed subgroup analysis was conducted to investigate the effect of different AS (sucralose, allulose, stevia, acesulfame potassium, and diet beverages).

**Table 1:** Results of the meta-analysis

Model	Statistic	Insulin	Glucose	C-Peptide	GLP-1	LDL	HDL	Triglyceride	HbA1c
	Number of studies (k)	5	8	3	4	5	5	5	3
Common Effect model	Hedges g (95%CI)	0.50 (0.19; 0.82)	0.16 (-0.05; 0.36)	-0.20 (-0.69; 0.29)	-0.14 (-0.58; 0.30)	0.01 (-0.16; 0.19)	0.11 (-0.07; 0.28)	-0.18 (-0.36; -0.00)	0.30 (0.06; 0.54)
	z	3.14	1.48	-0.80	-0.61	0.15	1.17	-1,97	2.44
Random Effects model	Hedges g (95% CI)	0.45 (-0.12; 1.01)	0.94 (-0.19; 2.06)	-0.20 (-0.69 0.29)	-0.14 (-0.58; 0.30)	0.01 (-0.16; 0.19)	0.11 (-0.07; 0.28)	-0.18 (-0.36; -0.00)	0.38 (-0.48; 1.24)
	z	1.56	1.64	-0.80	-0.61	0.15	1.17	-1.97	0.87
	tau^2^ (95% CI)	0.25 (0.00 3.18)	2.64 (0.87; 13.05)	0 (0.00; 4.75)	0 (0.00; 0.31)	< 0.00 (0.00; 0.58)	0 (0.00; 0.24);	0 (0.00; 0.02)	0.49 (0.08; 22.22)
Heterogeneity	tau (95% CI)	0.50 (0.00; 1.78)	1.54 (0.93; 3.61)	0 (0.00; 2.18)	0 (0.00; 0.56)	0.00 (0.00; 0.76)	0 (0.00; 0.49)	0 (0.00; 0.14)	0.70 (0.29; 4.71)
	I^2^ (95% CI)	64.1% (5.5%; 86.4%)	86.4% (75.4%; 92.5%)	0% (0.0%; 89.6%)	0% (0.0%; 84.7%)	0% (0.0%; 79.2%)	0% (0.0%; 79.2%)	0% (0.0%; 79.2%)	89.7% (72.2%; 96.2%)
	H (95% CI)	1.67 (1.03; 2.71)	2.72 (2.01; 3.66)	1.00 (1.00; 3.10)	1.00 (1.00; 2.56)	1.00 (1.00; 2.19)	1.00 (1.00; 2.19)	1.00 (1.00; 2.19)	3.11 (1.90; 5.11)

**Table 2:** Results of the Egger test for publication bias

Outcome	t-statistic	df	Bias estimate	SE (Bias)	tau^2^ (heterogeneity)	Publication Bias	Fail Safe N
Insulin	-0.33	3	-0.86	2.59	3.58	No	10
Glucose	2.73	6	3.70	1.36	3.84	Yes	40
C-Peptide	-1.06	1	-4.05	3.81	0.67	No	0
GLP-1	1.14	2	2.04	1.79	0.19	No	0
LDL	-0.83	3	-0.88	1.06	0.80	No	0
HDL	-1.21	3	-1.04	0.85	0.51	No	0
Triglycerides	1.02	3	0.52	0.51	0.18	No	1
HbA1c	0.27	1	1.72	6.34	18.04	No	6

### Quantitative synthesis/meta-analysis and evidence of effectiveness

**Figure 2:**
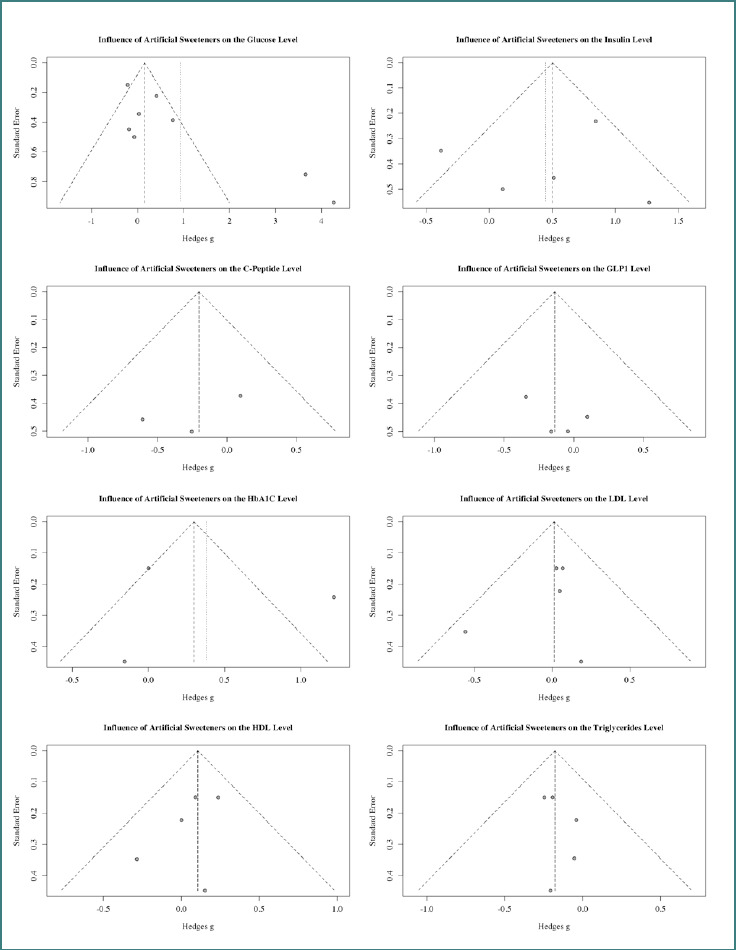
Panel plot of the funnel plots on the influence of artificial sweeteners

In sum, eight studies focused on the effect of AS on glucose. Three of them [17-19] reported increased glucose levels. In particular, Temizkan *et al*. [18] and Muilwijk *et al*. [19] reported very high increases of 4.26 and 3.66 standard deviations (SD) between the intervention (diabetic patients) and the control group (healthy patients). The other five studies showed mixed results of minor relevance. Also, the heterogeneity (86.4%) indicated a high amount of variance between the studies. A look at the panel plot (Figure 2) revealed an asymmetry in the effect of artificial sweeteners on glucose levels. Publication bias is possible, so the results should be interpreted with caution. The subgroup analysis (Tables 3 and 4) showed that the type of sweetener can affect glucose levels. In particular, stevia showed a difference between the intervention and control groups (0.66 SDs in the random-effects model). Also, heterogeneity is a big concern for those results. Five studies examined the effect of AS on insulin levels in patients with diabetes. Temizkan *et al*. [18] and Madjd *et al*. [20] found a difference in the level of insulin between diabetic and healthy patients of 1.27 and 0.84 SDs. This is supported in the common effects model (0.50), but not in the random effects model. All other studies reported mixed but not relevant effects. The heterogeneity (64.1%) was better than glucose but still very high. The funnel plot (Figure 2) is symmetric and does not indicate publication bias. Sucralose and the studies with diet beverages showed, in both models (Tables 3 and 4), a relevant effect of insulin level (1.2685 and 0.8437 SDs), and the common effects model showed a positive effect. However, there was also substantial variance across the mentioned studies.

**Table 3:** Common effects model for subgroups

Subgroup	Type of sweetener	k	Hedges g	95% CI	Q	I^2^
Glucose	Sucralose	2	-0.10	(-0.39; 0.19)	22.08	95.5%
	Allulose	2	-0.14	(-0.79; 0.52)	0.03	0.0%
	Stevia	2	0.66	(0.04; 1.28)	19.28	94.8%
	Diet Beverage	1	0.41	(-0.03; 0.85)	0.00	--
	Sucralose / Acesulfame K	1	0.77	(0.02; 1.53)	0.00	--
	Sucralose	1	12.69	(0.18; 2.35)	0.00	--
Insulin	Allulose	1	0.11	(-0.87; 1.09)	0.00	--
	Stevia	2	-0.05	(-0.60; 0.49)	2.43	58.8%
	Diet Beverage	1	0.84	(0.39; 1.30)	0.00	--
C-Peptide	Sucralose	1	-0.25	(-1.24; 0.73)	0.00	--
	Allulose	1	-0.61	(-1.51; 0.29)	0.00	--
	Sucralose / Acesulfam K	1	0.10	(-0.64; 0.83)	0.00	--
	Sucralose	1	-0.04	(-1.02; 0.94)	0.00	--
GLP-1	Allulose	1	-0.16	(-1.14; 0.82)	0.00	--
	Stevia	1	0.10	(-0.78; 0.97)	0.00	--
	Sucralose / Acesulfam K	1	-0.34	(-1.08; 0.40)	0.00	--
HbA1c	Sucralose	1	0.00	(-0.29; 0.29)	0.00	--
	Allulose	1	-0.16	(-1.03; 0.72)	0.00	--
	Diet Beverage	1	12.17	(0.74; 1.69)	0.00	--
	Diet Beverage	2	0.06	(-0.18; 0.31)	0.01	0.0%
LDL	Sucralose	1	0.03	(-0.27; 0.32)	0.00	--
	Allulose	1	0.19	(-0.69; 1.07)	0.00	--
	Stevia	1	-0.56	(-1.25; 0.13)	0.00	--
	Diet Beverage	2	0.16	(-0.08; 0.41)	0.78	0.0%
HDL	Sucralose	1	0.09	(-0.20; 0.38)	0.00	--
	Allulose	1	0.15	(-0.73; 1.03)	0.00	--
	Stevia	1	-0.29	(-0.97; 0.40)	0.00	--
Triglycerides	Diet Beverage	2	-0.15	(-0.39; 0.10)	0.32	0.0%
	Sucralose	1	-0.24	(-0.54; 0.05)	0.00	--
	Allulose	1	-0.21	(-1.09; 0.67)	0.00	--
	Stevia	1	-0.05	(-0.73; 0.62)	0.00	--

**Table 4:** Random effects model for subgroups

Subgroup	Type of sweetener	k	Hedges g	95% CI	tau^2^	tau
	Sucralose	2	19.30	(-2.46; 6.31)	95.75	30.94
Glucose	Allulose	2	-0.14	(-0.79; 0.52)	0.00	0.00
	Stevia	2	17.80	(-1.78; 5.33)	62.50	25.00
	Diet Beverage	1	0.41	(-0.03; 0.85)	--	--
	Sucralose / Acesulfame K	1	0.77	(0.02; 1.53)	--	--
	Sucralose	1	12.69	(0.18; 2.35)	--	--
Insulin	Allulose	1	0.11	(-0.87; 1.09)	--	--
	Stevia	2	0.02	(-0.85; 0.89)	0.24	0.48
	Diet Beverage	1	0.8437	(0.3888; 1.2986)	--	--
C-Peptide	Sucralose	1	-0.25	(-1.24; 0.73)	--	--
	Allulose	1	-0.61	(-1.51; 0.29)	--	--
	Sucralose / Acesulfam K	1	0.10	(-0.64; 0.83)	--	--
HbA1c	Sucralose	1	0.00	(-0.29; 0.29)	--	--
	Allulose	1	-0.16	(-1.03; 0.72)	--	--
	Diet Beverage	1	12.17	(0.74; 1.69)	--	--
	Sucralose	1	-0.04	(-1.02; 0.94)	--	--
GLP-1	Allulose	1	-0.16	(-1.14; 0.82)	--	--
	Stevia	1	0.10	(-0.78; 0.98)	--	--
	Sucralose / Acesulfam K	1	-0.34	(-1.08; 0.40)	--	--
	Diet Beverage	2	0.06	(-0.18; 0.31)	0.00	0.00
LDL	Sucralose	1	0.03	(-0.27; 0.32)	--	--
	Allulose	1	0.19	(-0.69; 1.07)	--	--
	Stevia	1	-0.56	(-1.25; 0.13)	--	--
	Diet Beverage	2	0.16	(-0.08; 0.41)	0.00	0.00
HDL	Sucralose	1	0.09	(-0.20; 0.38)	--	--
	Allulose	1	0.15	(-0.73; 1.03)	--	--
	Stevia	1	-0.28	(-0.97; 0.40)	--	--
Triglycerides	Diet Beverage	2	-0.15	(-0.39; 0.10)	0.00	0.00
	Sucralose	1	-0.24	(-0.54; 0.05)	--	--
	Allulose	1	-0.21	(-1.09; 0.67)	--	--
	Stevia	1	-0.05	(-0.73; 0.62)	--	--

Three studies looked at the effect of AS on C-peptide levels in patients with diabetes. All of them provided mixed results. Both the common and random effects models indicated a slightly negative effect (-0.20). The results were consistent (heterogeneity: 0%), so the studies are comparable. The funnel plot (Figure 2) is symmetric and does not indicate a publication bias. The subgroup analysis (Tables 3 and 4) also showed no effect of the different types of AS on C-peptide levels.

Four studies examined the influence of AS on GLP-1 levels in patients with diabetes. Three of them indicated a decrease in GLP-1 levels, and one showed an increase. None of those results are relevant, confirmed by the common and random effects model (-0.20). The results are consistent (heterogeneity: 0%), indicating the studies are comparable and have no design variance. The corresponding funnel plot (Figure 2) is symmetric, and there is no reference point for a publication bias. The test results for the subgroup analysis (Tables 3 and 4) revealed no effect of the individual sweetener type.

HbA1c was part of three studies, the least used outcome in the analysis. In this case, the study by Madjd *et al*. [20] showed a difference of 1.22 SDs between the intervention and control groups. This indicates a positive relationship between AS and HbA1c in patients with diabetes. The other two studies did not show relevant effects. The connection is supported in the common effects model (0.30), but not in the random effects model (0.38). What must be kept in mind is the high variance between the studies (heterogeneity: 89.7%), which is, of course, related to the low number of studies. The funnel plot (Figure 2) revealed no indication of a publication bias. In the subgroup analysis (Tables 3 and 4), the diet beverage group showed an effect on HbA1c (1.22), but it is unclear which sweeteners the authors used.

In five studies, the effect of LDL level was investigated. In summary, no relationship could be found. The results are mixed: four studies suggest a slightly positive influence, and one suggests a negative influence. The common and random-effect models (0.01) provided similar insights. The results are consistent (heterogeneity: 0%), which means that the studies are comparable and there is no variance. The funnel plot (Figure 2) shows no asymmetry, indicating a very low risk of publication bias. In the subgroup analysis (Tables 3 and 4), there is no indication that specific types of sweeteners influence LDL levels.

Five studies investigated the effect of AS on HDL. Nearly all of them reported an increase in the HDL level, except for Ajami *et al*. [14], who reported a slight decrease of -0.2850 SDs. A look at the common and random effects model reveals that both show the same value (0.11). This indicates a slight increase in the HDL level. All results are consistent (heterogeneity: 0%), so the studies are comparable, and the results are not due to variance across studies. The corresponding funnel plot (Figure 2) is symmetric and shows no indication of a publication bias. The subgroup analysis (Tables 3 and 4) provides the same insights as in the LDL analysis. No specific type of sweetener had a relevant influence on HDL levels.

Five studies examined the effect of AS on triglyceride levels. All of them indicated a decrease in triglyceride levels. This decline can also be seen in the common and random effects models, which both showed the same result (-0.18). The models indicate that more studies in this field should be conducted. All studies are consistent and comparable (heterogeneity: 0%). The funnel plot (Figure 2) is symmetric and provides no sign of a publication bias. The subgroup analysis (Tables 3 and 4) shows the same results. Every type of AS decreased triglyceride levels.

## Discussion

To address whether AS should be mandated in the nutrition plans of patients with diabetes, a systematic review and meta-analysis were conducted. As a result of a literature search according to the PRISMA guidelines [7], 22 studies have been included in this review. Those studies comprise 12 RCTs, 7 cross-sectional studies, 2 case-control studies, and 1 cohort study. Nearly all of them had a low to moderate risk of bias, except for three cross-sectional studies, which had a high risk of bias according to the JBI Critical Appraisal Tool [11]. The results of those included studies are pretty heterogeneous, and many focus on different outcomes. A look at the RCT studies reveals that the authors emphasized diabetic parameters like GLP-1 [17,18], glucose [14,17-23], insulin [14,18-21], or HbA1c [20,22,23]. Those studies produced mixed outcomes regarding these parameters, so a closer look into this topic is necessary. The same implication can be drawn from studies that focused on patients' lipid profiles. Some studies suggested that HDL, LDL, and triglyceride levels increased after consuming AS in patients with diabetes [20, 22-24], whereas Ajami *et al*. [14] reported a reduction. Regarding diet quality and weight management, two studies yielded contrasting results. Odegaard *et al*. [25] reported that switching from water to diet beverages has no effect on diet quality or energy intake, whereas Madjd *et al*. [20] found that drinking water leads to greater weight loss and a lower BMI. Future RCTs with larger sample sizes are needed to clarify the impact of AS on patients with diabetes. The cross-sectional studies were mainly done with questionnaires. Here, results indicated that the consumption of AS could lead to hypertension and obesity [26] and that people rarely knew about the effects that AS could have on their bodies [27]. The results of the case-control studies suggest that aspartame could affect the blood glucose and triglyceride levels [28] and that the risk for LADA was twice as high for patients with type 2 diabetes with the consumption of artificial sweeteners [15]. One of the most interesting study insights came from the only cohort study in this review. Campos *et al*. [16] found a positive connection between sucralose and gestational diabetes.

In the meta-analysis, two of the eight selected outcomes showed a relationship with AS intake in the common effects model. The levels of insulin and HbA1c increased compared to the healthy participants in those studies. These findings may be generalizable to adult patients with type 2 diabetes, as most included studies investigated this population. However, they might not directly apply to patients with type 1 diabetes or gestational diabetes, where insulin regulation differs. From a practical standpoint, this suggests that healthcare professionals should consider monitoring insulin levels more closely when recommending artificial sweeteners as sugar substitutes. Dieticians could, for instance, advise patients to observe postprandial glucose responses after switching to AS-containing products to avoid episodes of hypoglycemia.

However, measuring insulin can be quite difficult for patients with diabetes. Given their treatment, the timing of the measurement is quite important. If the time point differs across blood samples, the results could be biased. The HbA1c value, which measures long-term blood sugar control, is also a good indicator that the treatment of the diabetic illness is sufficient. If the intake of AS influences it, then the use of those substances in the nutrition of patients with diabetes needs to be questioned. This result also has practical implications for the design of individualized nutrition plans. If HbA1c levels are affected by AS consumption, clinicians may need to adjust treatment goals or patient education accordingly. On a broader level, these findings could inform updates to dietary recommendations and clinical guidelines for diabetes management.

Before this review, a literature search was conducted to define the topic. No meta-analysis was found during this research, indicating that, to the best of the author's knowledge, this is the first scientific work to examine the existing studies on AS in patients with diabetes. Therefore, this review provides an important step towards summarizing and quantifying the current evidence base. Even though the included studies were heterogeneous, the overall trends are representative of adults in industrialized countries, where artificial sweetener use is most prevalent.

The findings contribute to the growing debate about whether AS should be promoted as a safe and effective tool for glucose control or instead used with caution due to possible metabolic side effects.

However, the generalizability of the findings remains limited due to heterogeneity across study populations, diabetes types, treatment regimens, and exposure durations to artificial sweeteners. As a result, the observed effects should be interpreted cautiously, and the current evidence does not allow for definitive conclusions regarding long-term metabolic safety or clinical benefit. These limitations highlight the need for individualized dietary recommendations rather than universal guidance.

Existing evidence indicates that AS can help patients with diabetes stabilize their glucose levels, which is consistent with the results of this study [29]. Other articles focus on the risk of getting diabetes through the consumption of AS, with mixed results. Some studies suggest a relationship, others do not [30,31]. In 2014, a systematic review and meta-analysis failed to provide evidence for a link between AS and the development of diabetes. Instead, they thought that lifestyle factors and reverse causality could explain this connection [32].

The main objective of this work was to provide implications for clinical practice for doctors and nutritionists. Due to the ambiguous results, the author cannot give a clear recommendation for action. Nonetheless, the current results highlight that a “one-size-fits-all” approach is not appropriate. The effect of AS likely depends on individual metabolic profiles, duration of use, and overall diet composition. In practice, this means that clinicians and dietitians should personalize their recommendations, integrating AS use only when it clearly benefits glycemic control without increasing other metabolic risks.

For over 200 years, AS has been a big part of the everyday diet, including for patients with diabetes. In this timeframe, many studies and organizations have researched this topic. They mostly concluded that usage in a regular diet is safe for humans. The insights from studies of patients with diabetes since 2004 do not indicate a mandatory use of AS. This is in line with other reviews about the adverse side effects of AS, like a higher risk of cancer [33] or cerebrovascular diseases [34]. Individual factors (e.g., physical activity, high consumption of ultra-processed foods) and a well-thought use of AS should serve as the basis for developing nutrition guidelines for patients with diabetes.

An important group that was not explicitly examined in most of the included studies is individuals with prediabetes. This population represents a large and growing segment of those at risk of progressing to type 2 diabetes, and dietary modification is currently the primary recommended intervention. Understanding whether artificial sweeteners can improve glycemic control, maintain weight, or conversely accelerate metabolic dysregulation in prediabetes is of particular clinical relevance. Future studies should therefore include prediabetes participants to clarify whether AS could play a preventive or harmful role during this transitional metabolic state.

Future research should focus more on the effects of AS on patients with diabetes, especially regarding the consequences on their blood parameters. More studies with larger sample sizes are needed to provide qualitative and quantitative recommendations. This is also important from a public health perspective. As mentioned in the introduction, diabetes is one of the most common diseases worldwide, so many patients require specific dietary guidance.

Due to the rising number of products containing AS (e.g., fitness supplements or “high protein” products) and growing concerns about long-term health effects, the recommendations of the FDA and the European Food Safety Authority (EFSA) regarding the acceptable daily intake (ADI) may need to be revised. Future studies need to clarify the relationship with more patients, a longer observation period, and a specific focus on AS (not on lifestyle factors, diet quality, or energy intake).

The results of this systematic review and meta-analysis should be interpreted with some limitations in mind. Research and statistical work were conducted by a single person, so the literature review and interpretation of the outcomes may be biased by this one-dimensional approach. Also, the database search was limited because the focus was laid on articles available through a license from the Ludwig-Maximilians-University. Some authors have been asked to provide their studies, but have never granted access. It has to be considered that some papers with relevant information are not part of this review. The inclusion criteria for this review should be kept in mind when interpreting the results. Studies older than 20 years were excluded, and the authors focused on human studies with participants aged 18 years or older. Also, it was mandatory that every study be published in English. Sources from grey literature (e.g., Google Scholar) were not part of this review. Other data sources, aside from the mentioned one, were not part of this study.

The included studies covered different types of diabetes, including type 1, type 2, and gestational diabetes. The author intentionally did not restrict the inclusion criteria to a specific type of diabetes in order to capture a broader range of evidence. However, this approach may have contributed to heterogeneity in results, as the pathophysiology and treatment strategies differ substantially across these subgroups. Another limitation is that no inclusion or exclusion criteria were applied regarding the treatment of diabetic patients, such as insulin therapy or oral antidiabetic agents. This decision was made to avoid excluding potentially relevant studies. Nevertheless, different treatment regimens may have influenced metabolic parameters, such as glucose and insulin levels, which should be considered when interpreting the results.

Most studies attempted to eliminate potential confounders or adjust for them. Thus, it cannot completely rule out the possibility that synergistic effects (such as physical activity or other dietary factors) associated with AS consumption could influence the different outcomes. The study populations in all included articles were very small. The meta-analysis in this review focused on the variables mentioned because they are important for patients with diabetes. Other variables (e.g., systolic and diastolic blood pressure) were not included due to scarce data. However, a look at those outcomes could be interesting for future studies. Furthermore, BMI was not considered as an inclusion criterion, although it can significantly affect outcomes related to glucose metabolism and insulin resistance. Since BMI data were inconsistently reported across studies, it was not possible to analyze or control for this variable in the meta-analysis. Future research should address BMI as an important confounding factor when investigating the metabolic effects of artificial sweeteners.

Sensitivity analyses were not performed due to the relatively small number of studies and the heterogeneity of the included data. Conducting such analyses could have provided additional insights into the robustness of the results, but given the limited dataset, their interpretability would have been restricted. A last limitation is the short time frame of 4 months in which this review needed to be finished.

## Conclusion

This literature review and meta-analysis investigated the effect of AS on patients with diabetes and, to the author's knowledge, was the first of its kind. The research was done according to the PRISMA guidelines for systematic reviews. 22 studies were included in this paper with ambiguous results. The meta-analysis showed higher insulin and HbA1c associated with AS intake in the common-effects model, but these associations were not significant in the random-effects model, and heterogeneity was substantial. Therefore, the current evidence does not support a general or mandatory recommendation for artificial sweeteners in diabetes management. Individuals with prediabetes represent an important and growing group in whom dietary intervention is key, and future research should clarify whether artificial sweeteners help maintain glycemic control or may accelerate progression to diabetes. Further well-designed randomized controlled trials with larger sample sizes are needed to understand better the long-term metabolic and clinical effects of artificial sweeteners in both diabetes and prediabetes.

## Data Availability

The datasets used are available from the corresponding author upon reasonable request.
