# Supplementary material for: Artificial sweeteners and their implications for patients with diabetes: a systematic review and meta-analysis
Source: J Med Life. 2026 Jan;19(1):33–42. doi: 10.25122/jml-2025-0135 (PMC12973925; doi:10.25122/jml-2025-0135)
Supplement: Supplementary file 1 [file JMedLife-19-033-s001.pdf]

Supplementary File

Table S1. Subgroups and keywords

Subgroup	Keywords
Artificial sweeteners	artificial sweeteners<br>non-nutritive sweeteners<br>low-calorie sweeteners<br>sugar substitutes<br>aspartame<br>sucralose<br>saccharin<br>stevia<br>acesulfame potassium
Diabetes	diabetes<br>diabetes mellitus<br>type 1 diabetes<br>type 2 diabetes<br>T1D<br>T2D<br>gestational diabetes
Outcome	blood glucose<br>glycemic index<br>weight management<br>body mass index<br>BMI

Table S2. Inclusion and exclusion criteria

PICOS	Inclusion	Exclusion
Population (P)	• Studies including adults (≥18 years) diagnosed with any form of diabetes (e.g., type 1 diabetes, type 2 diabetes, gestational diabetes).<br>• Studies focusing on individuals at high risk of developing diabetes (e.g., pre-diabetes, metabolic syndrome).	• Studies focused on children or adolescents (<18 years).<br>• Studies on healthy individuals without diabetes or diabetes risk factors.<br>• Studies on animals
Intervention (I)	• Studies examining the use of AS (e.g., aspartame, sucralose, saccharin, stevia, acesulfame potassium, etc.) in food, beverages, or as a dietary supplement.<br>• Interventions that measure the impact of AS on diabetes-related outcomes (e.g., blood glucose levels, insulin sensitivity, HbA1c, weight management).	• Studies using natural sweeteners or sugar alcohols (e.g., honey, agave syrup, sorbitol, xylitol).<br>• Studies that did not specifically measure outcomes related to diabetes or glucose metabolism.
Comparison (C)	• Studies comparing AS to other dietary interventions (e.g., regular sugar, low-calorie diets).<br>• Studies comparing AS to a placebo or no intervention.<br>• Studies comparing the use of AS with healthy adults	• Studies without a clear comparison group.<br>• Studies comparing different forms of AS without considering their impact on diabetes outcomes.
Outcome (O)	• Studies reporting at least one relevant diabetes-related outcome, such as:<br>• Blood glucose levels (fasting, postprandial).<br>• HbA1c levels.<br>• Insulin resistance or sensitivity.<br>• Weight management (BMI, body weight).<br>• Incidence or progression of diabetes-related complications.	• Studies that did not report diabetes-related outcomes (e.g., focus solely on other metabolic outcomes like cholesterol or triglycerides without diabetes-related data).<br>• Studies that only reported subjective outcomes (e.g., taste preference) without any physiological or clinical measures.
Study design (S)	• RCTs<br>• Cohort<br>• Case-control<br>• Cross-sectional	• Case reports, case series, opinion pieces, and editorials.<br>• Animal studies or in vitro studies.<br>• Systematic reviews and meta-analyses

© 2025 by the authors. This article is an open access article distributed under the terms and conditions of the Creative Commons Attribution (CC BY 4.0) license.

Table S3. Results of the quality assessment process

Study title	Type	Risk of bias
Sucralose enhances GLP-1 release and lowers blood glucose in the presence of carbohydrate in healthy subjects but not in patients with type 2 diabetes [18]	RCT	Low
Investigating the use and awareness of artificial sweeteners among diabetic patients in Bangladesh [26]	Cross-sectional study	Moderate
Short-term effects of allulose consumption on glucose homeostasis, metabolic parameters, incretin levels, and inflammatory markers in patients with type 2 diabetes: a double-blind, randomized, controlled crossover clinical trial [21]	RCT	Low
The effect of substituting water for diet beverages on diet quality in people with type 2 diabetes: the study of drinks with artificial sweeteners (SODAS), a randomized trial [25]	RCT	Moderate
The effect of substituting water for diet beverages on blood pressure and lipids in people with type 2 diabetes (T2D): the study of drinks with artificial sweeteners (SODAS) [24]	RCT	Moderate
The entero-endocrine response following a mixed-meal tolerance test with a non-nutritive pre-load in participants with pre-diabetes and type 2 diabetes: A crossover randomized controlled trial proof of concept study [19]	RCT	Low
Effect of artificial sweeteners on insulin resistance among type-2 diabetes mellitus patients [35]	Cross-sectional study	High
Effect of replacing sucrose in beverages with nonnutritive sweetener sucralose on cardiometabolic risk factors among Asian Indian adults with type 2 diabetes: a 12-week randomized controlled trial [22]	RCT	Low/Moderate
Sweetened beverage intake and risk of latent autoimmune diabetes in adults (LADA) and type 2 diabetes [15]	Case-control study	Low
Impact of long-term cyclamate and saccharin consumption on biochemical parameters in healthy individuals and type 2 diabetes mellitus patients [36]	Cross-sectional study	High
Awareness of health effects of aspartame among diabetic patients [37]	Cross Sectional Study	High
A pilot study on the efficacy of a diabetic diet containing the rare sugar d-allulose in patients with type 2 diabetes mellitus: a prospective, randomized, single-blind, crossover study [23]	RCT	Low/Moderate
Effects of stevia on glycemic and lipid profile of type 2 diabetic patients: a randomized controlled trial [14]	RCT	Low
Beneficial effects of replacing diet beverages with water on type 2 diabetic obese women following a hypo-energetic diet: a randomized, 24-week clinical trial [20]	RCT	Low
Relationship between sucralose consumption and serum concentration of glycosylated hemoglobin in people with type 2 diabetes mellitus without complications [38]	Cross-sectional study	Moderate
Knowledge, attitude, and practice toward non-nutritive sweeteners among the population with reduced sugar intake requirement [27]	Cross-sectional study	Moderate
Association between consumption of non-nutritive sweeteners and gestational diabetes mellitus in Chilean pregnant women: A secondary data analysis of the CHiMINCs-II cohort [16]	Cohort study	Low
Effects of diet soda on gut hormones in youths with diabetes [17]	RCT	Moderate
The relationship between diet soda soft drinks, artificial sweeteners, and diabetic retinopathy: a cross-sectional study [39]	Cross-sectional study	Moderate
The effect of substituting water for diet beverages on CGM metrics in people with type 2 diabetes (T2D): the study of drinks with artificial sweeteners (SODAS) [40]	RCT	Moderate
The effect of diet beverage intake on measures of diabetes control: a pilot study [41]	RCT	Low/Moderate
Effectiveness of aspartame on insulin, triglycerides, and blood glucose concentration in adult type 2 diabetic patients [28]	Case control study	Moderate

© 2025 by the authors. This article is an open access article distributed under the terms and conditions of the Creative Commons Attribution (CC BY 4.0) license.

Figure S1. Forest plot of all outcomes

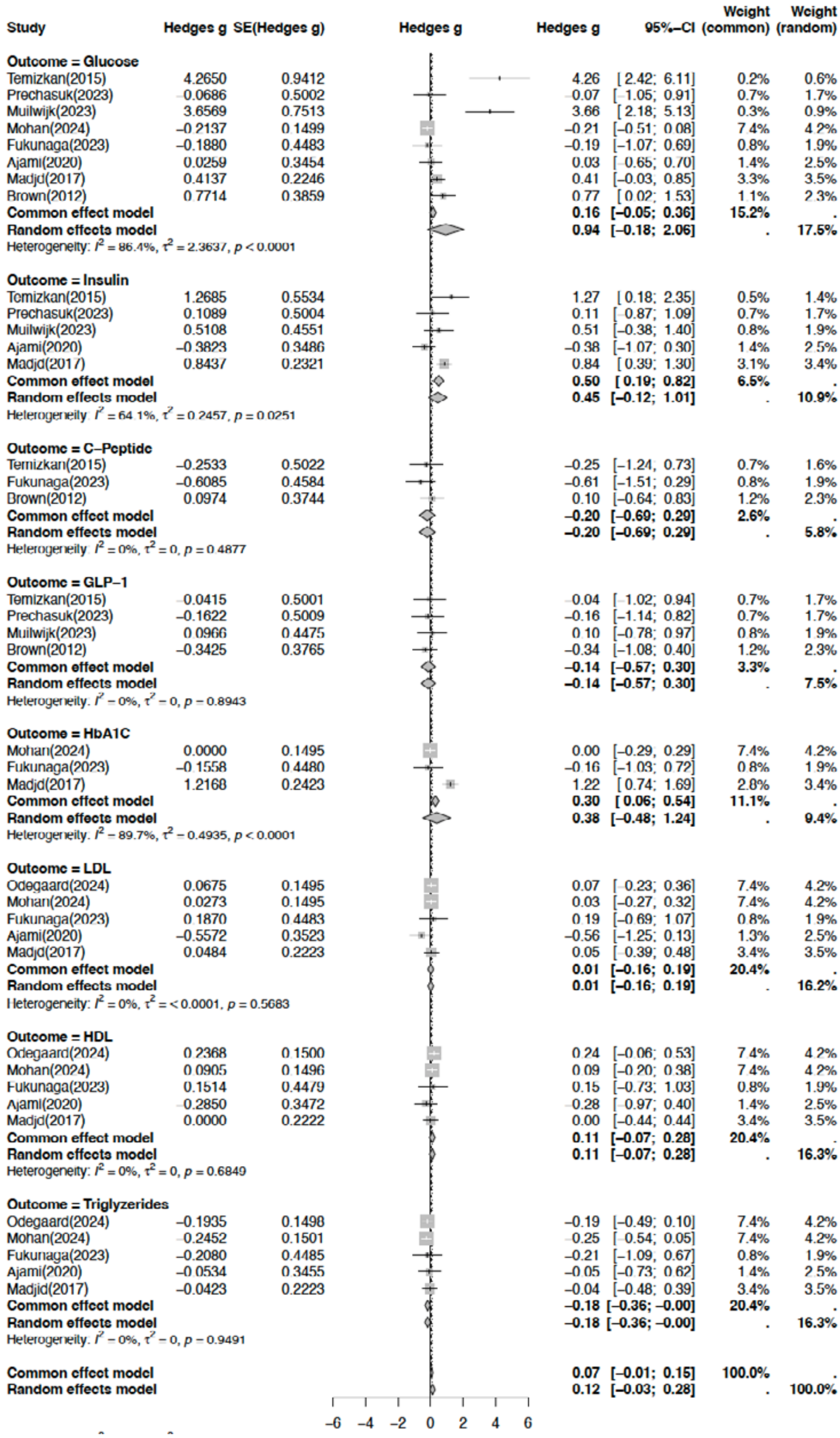

© 2025 by the authors. This article is an open access article distributed under the terms and conditions of the Creative Commons Attribution (CC BY 4.0) license.
